# Development, Implementation, and Compliance of Treatment Pathways in Radiation Medicine

**DOI:** 10.3389/fonc.2013.00105

**Published:** 2013-05-06

**Authors:** Louis Potters, Jadeep Raince, Henry Chou, Ajay Kapur, Daniel Bulanowski, Regina Stanzione, Lucille Lee

**Affiliations:** ^1^Department of Radiation Medicine, North Shore-LIJ Cancer Institute, Hofstra North Shore-LIJ School of MedicineNew Hyde Park, NY, USA

**Keywords:** radiation oncology, value-based medicine, care pathways, protocols

## Abstract

**Introduction:** While much emphasis on safety in the radiation oncology clinic is placed on process, there remains considerable opportunity to increase safety, enhance outcomes, and avoid *ad hoc* care by instituting detailed treatment pathways. The purpose of this study was to review the process of developing evidence and consensus-based, outcomes-oriented treatment pathways that standardize treatment and patient management in a large multi-center radiation oncology practice. Further, we reviewed our compliance in incorporating these directives into our day-to-day clinical practice.

**Methods:** Using the Institute of Medicine guideline for developing treatment pathways, 87 disease specific pathways were developed and incorporated into the electronic medical system in our multi-facility radiation oncology department. Compliance in incorporating treatment pathways was assessed by mining our electronic medical records (EMR) data from January 1, 2010 through February 2012 for patients with breast and prostate cancer.

**Results:** This retrospective analysis of data from EMR found overall compliance to breast and prostate cancer treatment pathways to be 97 and 99%, respectively. The reason for non-compliance proved to be either a failure to complete the prescribed care based on grade II or III toxicity (*n* = 1 breast, 3 prostate) or patient elected discontinuance of care (*n* = 1 prostate) or the physician chose a higher dose for positive/close margins (*n* = 3 breast).

**Conclusion:** This study demonstrates that consensus and evidence-based treatment pathways can be developed and implemented in a multi-center department of radiation oncology. And that for prostate and breast cancer there was a high degree of compliance using these directives. The development and implementation of these pathways serve as a key component of our safety program, most notably in our effort to facilitate consistent decision-making and reducing variation between physicians.

## Introduction

The correct time to adopt new technology and treatment options has always posed a clinical challenge. Given the increasing complexity of cancer treatment techniques, there is also potentially a negative impact on patient safety, treatment effectiveness, and efficiency of treatment delivery. These concerns have prompted an effort to standardize our delivery of radiation therapy based on best practices determined by published evidence and consensus. Treatment pathways and clinical guidelines aimed at creating more uniform and standard measures of care are increasingly utilized to address the need for care optimization. Clinical guidelines such as those prepared by the National Comprehensive Cancer Network (NCCN)^1^ have been developed as decision trees that broadly define oncologic care. Nevertheless, for radiation oncology, these guidelines fail to provide details that are relevant to the field. The Radiation Therapy Oncology Group (RTOG)^2^ treatment protocols offer detailed treatment pathways in radiation oncology; yet, only reflect a small portion of everyday clinical care and are rarely used outside of clinical trials. Thus, treatment pathways need to outline and standardize care details that match evidence-based outcomes and which offer guidance and consistency for the detailed and obtuse process of radiation planning, delivery, and clinical management.

The institution of treatment pathways, especially at a large, multi-center academic practice, faces many challenges. The most obvious challenge includes physician preference based on individual training and clinical experience. Even when physicians agree on a specific treatment protocol, significant variability may still arise in the delineation of the Clinical Target Volume (CTV) magnitude or relative target location. (Batumalai et al., 2011) Another factor subject to variability is the reporting of doses and outcomes from studies, as highlighted by the recent Quantitative Analysis of Normal Tissue Effects in the Clinic (QUANTEC)^3^, making the task of generating dose-response curves from data formidable (Emami et al., 1991). While substantial efforts toward standardizing treatment assessments in regards to toxicity have been made by the National Cancer Institute toward the Common Terminology Criteria for Adverse Events (CTCAE)^4^ and by the European Organization for Research and Treatment for Cancer (EORTC) for late effects using the Late Effects Normal Tissue Task Force subjective, objective, management, and analytic criteria (LENT-SOMA) (Pavy et al., 1995) there remains a lack of uniform and consistent integration of these metrics by the radiation community at large.

This study addresses the process of developing evidence and consensus-based, outcomes-oriented treatment pathways that incorporate the standardization of treatment algorithms and assessments into a large, multi-center academic radiation oncology practice. We reviewed our compliance reflecting physician acceptance and incorporation of the treatment pathways into our day-to-day clinical practice. Our primary analysis reviews how commonly used treatment pathways, for prostate cancer and early-stage breast cancer, comply with dose and fractionation scheme. Ultimately, adherence to dose constraints may correlate with morbidity and outcomes data. In this way, we hope to assure our patients of appropriate therapy delivered with recognized and accepted approaches.

## Materials and Methods

Starting in 2007, the Department of Radiation Medicine at North Shore-LIJ Health System began the process of developing consensus-based treatment pathways based on the Institute of Medicine (IOM) outline for guideline development^5^ (Institute of Medicine Outline for Guideline Development in Appendix).

A Pathways Committee was formed consisting of members from all disciplines within the department (including radiation oncology physicians, physicists, dosimetrists, therapists, and nurses), with the stipulation that all members be current employes of the Health System and not possess any conflicts of interest. The committee was charged with creating treatment pathways based on a standard outline and incorporating evidence-based treatment options to define all aspects of radiation therapy. Allowed avenues of evidence included recognized national or society guidelines, results of randomized controlled trials, phase I and II clinical studies, and current on-going cooperative group trial pathways. The pathways are broken down by disease site and stage, and include the treatment pathway, which is composed of details on the prescription, including treatment site, technique, modality, prescribed dose, daily fraction dose, number of fractions, and treatment schedule; treatment planning parameters, inclusive of contouring definitions, and dose constraints to all targets and normal tissue; and treatment assessments, which include on-treatment nursing instructions, and survivorship details including a follow-up schedule and post treatment testing. An example of a pathway is provided for external beam radiation therapy for the breast (Example of External Beam Radiation Therapy Protocol for Breast Cancer in Appendix).

Each pathway was then distributed to the faculty and staff within the Department for feedback, and a second final review by the committee was performed prior to inclusion in our electronic medical record (EMR) (MOSAIQ, Elekta). All pathways are re-reviewed and adapted to ensure they continued to reflect evolving evidence and clinical issues if they were to arise. After a treatment pathway and assessment standard has been completed and approved, a billing guideline is established using Current Procedural Terminology (CPT)^6^ that is linked to each Pathway. These pathways can then be chosen and linked in the EMR for the treatment of any patient and subsequently the compliance of each pathway can be audited.

The pathways were also re-reviewed in 2010 and updated based on the recommendations of the QUANTEC (see text footnote 3) that summarized dimensional dose/volume/outcome data for many organs that were defined in terms of normal tissue dose/volume tolerances (Fowler et al., 1963; Deasy, 2010).

Each physician has latitude to select any treatment pathway from a library or to write their own treatment prescription for their patients. The intent is to create treatment pathways that are applicable to the majority of patients and that may require only minimal editing. Therefore, the physician still has allowance, albeit limited; to personalize the patient’s care based on what they believe is optimal treatment when using a pathway from the library.

Analysis from our EMR for breast and prostate cancer was performed to assess compliance in incorporating these directives into our day-to-day clinical practice. For quantitative and compliance analysis, we mined data from our EMR from January 1, 2010 through February 2012 for patients with breast and prostate cancer using the CPT code library. Compliance was then determined by matching the number of fractions given for each prescription to the chosen treatment pathway for each patient. Non-compliant patient charts were individually reviewed during routine weekly chart rounds.

## Results

Between 2008 and 2010, a total of 85 treatment pathways were developed and implemented within the EMR of our department (Table 1 and Example of External Beam Radiation Therapy Protocol for Breast Cancer in Appendix).

**Table 1:** **Current library of treatment pathways in use at North Shore-LIJ Health System**.

Class	Pathway
Breast	Breast Canadian tangents supine
Breast	Breast Canadian tangents prone
Breast	Breast_standard tangents supine
Breast	Breast standard tangents prone
Breast	Breast tangents nodes and boost
Breast	Breast_postmastectomy
Breast	Breast RTOG_1005_standard boost
Breast	Breast RTOG_1005_concurrent boost
Breast	Breast partial breast irradiation
GU	Prostate EBRT bat
GU	Prostate EBRT calypso
GU	Prostate EBRT IGRT
GU	Prostate EBRT + brachy
GU	Prostate nodes EBRT alone
GU	Prostate nodes EBRT + BRACHY
GU	Prostate brachytherapy
GU	Prostate post_prostatectomy
GU	Prostate RTOG 0815 EBRT alone
GU	Prostate RTOG 0815 brachy + EBRT
GU	Prostate 0924 whole pelvis
GU	Prostate 0924 prostate alone
GU	Bladder definitive chemort
GU	Bladder palliative
GU	Seminoma paraaortics
GU	Penis definitive chemort
GU	Prostate salvage brachytherapy
GYN	cervix definitive
GYN	CERVIX postop
GYN	Endometrial postop
GYN	Endometrial hdr_brachy
GYN	Endometrial definitive
Head and neck	HN_definitive IMRT + chemo
Head and neck	HN definitive IMRT alone
Head and neck	HN_postop IMRT
Head and neck	HN dahanca
Head and neck	Larynx early-stage
CNS	Brain GBM RTOG
CNS	Brain GBM EORTC
CNS	Brain glioma low grade
CNS	Brain meningioma_malignant
CNS	Brain meningioma atypical
CNS	Brain_pituitary
CNS	Brain metastatic 3000
CNS	Brain metastatic 3750
CNS	Brain PCI SCLC
CNS	Brain CNS lymphoma
CNS	Brain PEDS
CNS	Craniospinal
GI	Anal chemort
GI	Esophagus definitive chemort
GI	Gastric post OP chemort
GI	Pancreas_definitive chemort
GI	Pancreas neoadj_adjuvant
GI	Rectum_preop chemort
GI	Liver microspheres
Lung	Lung_NSCL chemort
Lung	Lung NSCL RT alone
Lung	Lung NSCL preop chemort
Lung	Lung NSCL postop RT
Lung	Lung SCLC chemort
SRS and SBRT	SRS AVM
SRS and SBRT	SRS_brain metastasis
SRS and SBRT	SRS_brain met large
SRS and SBRT	SRS_trigeminal_neuraglia
SRS and SBRT	SRS_acoustic_schwannoma
SRS and SBRT	SBRT_lung central
SRS and SBRT	SBRT lung peripheral
SRS and SBRT	SBRT_spine cervical
SRS and SBRT	SBRT spine thoracic
SRS and SBRT	SBRT spine lumbar
SRS and SBRT	SBRT prostate
SRS and SBRT	SBRT liver
SRS and SBRT	SBRT pancreas
Lymphoma	Hodgkins_bulky
Lymphoma	Hodgkins_low_risk
Lymphoma	Non_Hodgkins
Sarcoma	Sarcoma
Skin	Skin_basal_squamous
Skin	Skin_melanoma
Bone	Bone metastasis
Bone	Heterotopic bone
IVBT	18.4 Gy
IVBT	23.0 Gy
TBI	Adult
TBI	Protocol

Prior to the use of standardized treatment pathways, retrospective analysis of EMR data revealed that there was significant variability in the choice of total dose and dose per fraction. Between 2006 and 2008, seven different doses were used to treat the whole breast for stage I and II breast cancer. A total of 15 different boost doses were used during the same period. Further analysis of a period during which the pathways were implemented was performed and identified 155 patients (January 2011–February 2012) treated with stage I or II cancer of the breast and 294 patients (January 2010–March 2011) treated for clinically confined cancer of the prostate. The use of a specific treatment pathway versus an *ad hoc* prescription for these diagnoses was 100%. For each of these diagnoses, the overall compliance for the selection of either a breast or prostate directive was found to be 97 and 99% respectively (Table 2). The reason for non-compliance proved to be either a failure to complete the prescribed care based on grade II or III toxicity (*n* = 1 breast, 3 prostate) or patient elected discontinuance of care (*n* = 1 prostate) or the physician chose a higher dose for positive/close margins (*n* = 3 breast).

**Table 2:** **Compliance of prostate and breast cancer therapy**.

Prostate pathways	Number of cases	Percent usage (*N* = 292) January 2010–March 2011
Prostate EBRT	124	42
Prostate EBRT + brachy	25	9
Prostate brachytherapy	84	29
Prostate post_prostatectomy	30	10
prostate RTOG 0815 EBRT alone	8	3
Prostate RTOG 0815 brachy + EBRT	12	4
SBRT prostate	5	2
Non-compliant (does not use any of the above pathways)	4	1

**Breast pathways**	**Number of cases**	**Percent usage (*N* = 155) January 2011–February 2012**

Breast brachytherapy (mammosite)	12	8
Breast Canadian tangents (prone or supine)	57	37
Breast standard tangents (prone or supine)	58	37
Breast tangents nodes + boost	6	4
Breast post mastectomy	14	9
Breast RTOG_1005_standard boost	3	2
Non-compliant (does not use any of the above pathways)	5	3

## Discussion

This study demonstrates that consensus and evidence-based treatment pathways can be developed for a multi-center department of radiation oncology and implemented with high degrees of compliance. Moreover, hurdles such as physician bias and resistance to conformity can be overcome by offering pathways that have been rigorously researched and vetted. The utilization of these treatment pathways serves as a key component of our safety program. And while the pathways are not designed to eliminate the role of physician decision-making nor that of peer review, they do serve as a means of creating standard approaches for a specialty that offers a wide variety of subjective treatment options that can often lead to misunderstanding and confusion.

Safety in radiation oncology involves many aspects of the care continuum. Our safety program includes six-sigma mechanics, quality checklists, and interlocks defined as the “No Fly” policy (Kapur and Potters, 2012; Potters and Kapur, 2012). Our department initiates patient treatments based on timed activities for each event outlined within a given treatment plan. When a particular task is completed, it is checked off the quality checklist, which then triggers the next task to appear on the checklist. This system ensures that tasks are taking place on time, within the correct timeframe, and stops are in place so that nothing proceeds beyond an uncompleted task. Direct input and transparency from all aspects of the department (including nursing, dosimetry, and physics) when developing the pathway has allowed for a much better understanding of key issues that may be encountered when dealing with both routine and complex cases and has improved overall efficiency, compliance and resource utilization. Essentially, these pathways serve as a bridge between the medical decision-making process and the technical process of treatment delivery, providing a working foundation for the entire patient experience. Implementation of the treatment pathways within the context of our safety program helps to standardize the types of checklist items and the process of completion of those items. Furthermore, the rationale for these pathway is based upon the recommendations proposed by the Institute for Safe Medical Practices (ISMP)^7^, a federally certified non-profit organization devoted to patient safety in healthcare. According to the ISMP, standardization of care as outlined by the treatment pathway is ranked third behind Forcing Functions and Computerization in terms of effectiveness for hazard mitigation, and thus, for increasing patient safety.

The treatment pathways provide the foundation for a physician to manage a treatment plan that is both evidence and consensus based. They provide the prescription, which comprises the dose and fractionation, machine energy, and treatment technique for that particular treatment as well as detailed instructions on how to manage the patient during and after treatment. Utilization of treatment pathways facilitates more consistent decision-making and reduces variation between physicians in the management of patients with similar diagnoses. One argument against the creation and use of treatment pathways is the potential loss of autonomy in offering patients personalized care. Further, it is argued that physicians prefer to detail treatment based on how they were trained and on past experience. Nonetheless, we observed a high degree of compliance attributable to providing physicians with a range of options that allowed for personalization, yet remained within our guidelines for best evidence and consensus-based treatment. Compliance was also driven in part by the collaborative nature of the directives’ development as well as the safety advantage of using a care pathway familiar with the department staff. Having implemented these pathways directly into the EMR allows for the ease of selecting and optimizing treatment parameters for each patient. Care pathways created on the fly run the risk of being treated in a way that is either not communicated effectively to the rest of the health care team or in a manner that does not keep with current evidence. Most interestingly, the attempt (by author LP) to expand the directives to other departments in a collaborative manner with additional input failed, as physicians felt they would lose their autonomy by the (consensus-based) process and by using standardized treatment pathways.

The use of these pathways has not obviated the role of peer review, such as chart rounds. Nevertheless, it has changed chart rounds by focusing on those cases treated in a manner that differs from the treatment pathways. These discussions often provide opportunity to review and consider updates to the current pathways. Peer review also allows us to explore automated metrics that measure conformance in various aspects of treatment pathway-based care such as from physician, planner/physicist, nursing, follow-up, and billing. This should ultimately enhance the value of peer review and provide constant feedback that provides opportunity to hone the quality of the pathways.

The most recent IOM update of standards for developing trustworthy Clinical Practice Guidelines, with which our process for developing our pathways closely complies, calls for transparency, management of conflict of interest, creation of a multidisciplinary group, systematic reviews, evidence rating, articulation, external review, and updating. In the current study we failed to have an external review. In an effort to further enhance the process and to explore opportunities for external review, we are currently working with several other departments, such as medical oncology, on validation of our created pathways, as well as the possibility of standardizing care with other partnered institutions. Our inability to meet the IOM standard for external review is the direct result of skepticism from other institutions that consensus can be reached or that it is even meaningful (personal communication LP). Individual physicians bring biases from past clinical experience and more generally from where they trained. Often a physician will prescribe a treatment solely based on these biases – even if that treatment has not become the standard of care within the community. Overcoming this bias within our department itself proved to be most difficult when attempting to establish consensus on a given pathway where there was a lack of evidence for the proper treatment plan. However, our report citing the high rates of compliance in our academic clinic may serve as a catalyst for other departments to consider pathways as a viable alternative to a more subjective approach and participate in the process of creating more universal standards of care. If a more generalized consensus can be reached across several departments and implemented, this could have a profound effect on the safe delivery of radiation, especially since traditional guidelines present appropriateness of treatment, but often do not offer detailed therapy directives. The training of physics staff and therapists could become more uniform and the expectations of clinical care, such as nursing, would be more clearly defined. This would also have the added benefit of creating a larger base of patients that would have been treated in the same way, with the outcomes of these treatments therefore becoming more meaningful. Clearly we are not the first institution to have implemented internal treatment pathways, yet our process of creating and our overall compliance to these pathways serves as a model for other departments considering this change.

These pathways also offer a cost-effective, peer reviewed alternative to the guidelines of Radiation Oncology Business Management (ROBM) companies. ROBM companies have been increasingly dictating care as an effort to control the “cost of care” as a value-add to the managed care industry. Their efforts utilize the granularity of the CPT code process. The “black box” methodology of ROBM guideline development is generally framed primarily with cost containment in mind. Therefore, the focus is on minimally acceptable care standards for any given disease. Furthermore, the for-profit ROBM pathways are designed with the intent of managing resource utilization and cost associated with emerging technologies for radiation therapy – which is in direct contrast to the development and use of our pathways. As such, the obvious side effect of the ROBM’s increased market penetration is the development and mandated use of pathways with the explicit purpose of mitigating expense on a per-patient basis.

One unintended but key finding in our current study was the identification of how directives are used based on aggregate care. Upon institution of our pathways, there was no discussion on resources or costs associated with the different treatment options whatsoever – the selection of each pathway was at the discretion of the attending physician and based solely on selecting an appropriate pathway given an individual patient’s characteristics. Our high percentage use of Canadian fractionation for breast cancer (which is considered a less expensive option for the treatment of breast cancer) has been shown to drive down the aggregate cost of treating the disease – without compromising the quality of care for each individual patient since each treatment option was selected solely on the basis of providing the most effective, highest quality treatment for each patient. For instance, the average Medicare payment for breast cancer based on Directive Compliance was $10,888 compared to $14,698 payment for on standard (long course) treatment. The directive payment is 74% of long course treatment. Unlike ROBMs that manage individual cases by limiting resources per patient, the development and use of multiple care pathways does not discriminate the resources needed for any given patient necessary for evidence-based care, yet also seems to create an environment that decreases the overall cost of care.

In conclusion, we have developed an approach of directive development and implementation that demonstrates high degrees of compliance that also offers cost-effective care. What started out as, and remains, a key component of our safety program has afforded our physicians the opportunity to personalize care for their patients while also being assured that the care they deliver is both the safest and most effective for a given disease. We hope to work with other institutions in establishing external validation of our pathways and hopefully develop consensus that will create the impetus for transparent, evidence-based radiation therapy.

## Conflict of Interest Statement

The authors declare that the research was conducted in the absence of any commercial or financial relationships that could be construed as a potential conflict of interest.

## Footnotes

^1^http://www.nccn.org/professionals/physician_gls/f_guidelines.asp

^2^http://www.RTOG.org

^3^http://www.aapm.org/pubs/QUANTEC.asp

^4^http://ctep.cancer.gov/protocolDevelopment/electronic_applications/ctc.htm

^5^www.iom.edu/~/media/Files/Report%20Files/2011/Clinical-Practice-Guidelines-We-Can-Trust/Clinical%20Practice%20Guidelines%202011%20Report%20Brief.pdf

^6^https://catalog.ama-assn.org/Catalog/cpt/cpt_home.jsp

^7^https://www.ismp.org/orderforms/reporterrortoismp.asp

## Appendix

### Institute of medicine outline for guideline development

1. Establishing transparency
2. Management of conflict of interest
3. Guideline development group composition
4. Clinical practice guideline-systematic review intersection
5. Establishing evidence foundations for and rating strength of recommendations
6. Articulation of recommendations
7. External review
8. Updating

### Example of external beam radiation therapy protocol for breast cancer

.BREAST, STANDARD TANGENTS SUPINE

PRESCRIPTION

Rx site: breast _L/_R   (CHOOSE LATERALITY)

Technique: 3D conformal

Modality: 6–23 MV

Dose specified: as per plan

Rx dose: 5000 cGy

Fraction dose: 200 cGy

# Fx’s/pattern: 25/once daily

ON TREATMENT

Filming: tangents including entire field prior to first treatment, then weekly alternate medial and lateral tangent (planned field only, entire field not necessary).

Nanodots Frequency: first day, then weekly, five to six dots total

Nanodots Placement: setup patient to treatment isocenter. Rotate the gantry to 0°, turn on the field light, and place the dot on the central axis. Cover with 5 mm bolus.

2^nd^ Rx site: breast boost _L/_R (CHOOSE LATERALITY/OPTIONAL)

Technique: enface

Modality: 6–8 MeV

Dose specified: 90–100%

Rx dose: 1000 cGy

Fraction dose: 200 cGy

# Fx’s/pattern: 5/once daily

ON TREATMENT

Nanodots Frequency: first day, one dot total.

Nanodots Placement: center of electron field. DO NOT cover with bolus.

SIMULATION INSTRUCTIONS:

SUPINE

Preparation: no specific preparation required.

Position: supine on breast board with appropriate angle for upper torso, ipsilateral arm extended above head; contralateral arm at side; the patient’s midline (medial biangulation tattoo) is set with the *X*-axis laser at zero.

Devices: breast board, knee pillow, wires, BBs.

Procedures: mark lumpectomy scar and drain sites with wire, place wire at 2 cm below the inframammary margin.

CT scan: scan from sternal notch to 4 cm past inframammary margin.

Tattoos: total four – two medial and lateral BBs at approximate center of treatment plane, approximately 2 cm inferior to inframammary fold.

SUPINE BOOST at LIJ using Clarity ultrasound

Preparation: enter patient into the Clarity system.

Prior to CT scan, zero the couch Longitude at the biangulation plane to register the lumpectomy cavity origin.

CT scan: scan entire region as for breast tangents.

Obtain measurement from CT on slice that shows the lumpectomy cavity, from the skin surface down to the chest wall in order to set gain correctly.

Ultrasound: bring patient/table back to original longitude/zero position.

Remove wire and ultrasound area using steady, light pressure across the scar region.

Send scan to appropriate planning location and to Clarity.

Isocenter/tattoos: The origin sets the biangulation tattoos for the tangents and the boost will be derived from this origin. Same tattoos for the breast tangents.

PLANNING:

Technique: 3D conformal (forward planning).

Target definition:

MD contour: boost volume, heart for left-sided breast, mark four borders with points as per guidelines below.

Medial: mid-sternum.

Lateral: mid-axillary line.

Superior: base of clavicular head.

Inferior: 2 cm below the inframammary fold.

Boost CTV/PTV: MD will contour the cavity = CTV, then create PTV with 5–10 mm margin, cutout will be made with 5 mm beyond PTV.

Breast CTV/PTV: defined as 95% isodose line (by planner).

Heart (Left-sided treatment): contours should begin at the level of the bifurcation of the pulmonary artery into left and right branches, include great vessels, pericardium.

Target goals: dose constraints for planning includes all structures in DVH:

Breast CTV/PTV: D95 = 95%

Max dose ≤107%

Boost CTV/PTV: D95 = 95%

Max dose ≤115%

Ipsilateral lung: ideal V_20Gy_ ≤15%; acceptable V_20Gy_ ≤20%

Ipsilateral lung: ideal V_10Gy_ ≤35%; acceptable V_10Gy_ ≤40%

Ipsilateral lung: ideal V_5Gy_ ≤50%; acceptable V_5Gy_ ≤55%

Contralateral lung: ideal V_5Gy_ ≤10%; acceptable V_5Gy_ ≤15%

Heart (left-sided treatment): ideal V_20Gy_ ≤5%; acceptable V_25Gy_ ≤5%

Heart (left-sided treatment): ideal V_10Gy_ ≤30%; acceptable V_10Gy_ ≤35%

Heart mean dose: ideal ≤400 cGy; acceptable ≤500 cGy.

NURSING INTERVENTIONS

Consultation/education: document a complete evaluation including physical examination, review of systems, baseline vital signs, and blood work within 1 month of starting treatment. Complete patient education including simulation, filming, treatment, and possible side effects and management of these side effects. Review skin care and provide resources for obtaining skin care products.

After consultation: review QCL items needed and schedule items when appropriate, e.g., HCG at simulation, EKG for pacemaker at first treatment.

On treatment: start skin care treatment on or before day 1. Assess patient and document weekly during OTV and more often when needed, e.g., escalating side effect, pain management.

Laboratory: HCG at or prior to simulation for premenopausal patients, CBC ×1 during treatment.

Completion: on or prior to last treatment, provide patient with written discharge instructions.

FOLLOW-UP/SURVIVORSHIP

PTE at 1 month: ensure resolution of acute side effects, radiation dermatitis.

Follow-up: every 3–6 months afterward for 2 years, then every 6–12 months, then consider discharge to oncologist/PMD after 5 years.

Mammography annually: document report and order if not done.

Co-management: surgical oncologist, medical oncologist.

CPT CODE SETS: see Documents “Coding Outline.”

REFERENCES AND DISCUSSION

Patient selection: patients are candidates for breast conservation after lumpectomy for Tis, T1, T2 tumor, and occasionally T3 if negative margins can be achieved, with negative sentinel nodes/axillary node dissection. Patients who are 70 years old and older with stage I, ER (+) tumors and clinically negative lymph nodes, taking tamoxifen or an aromatase inhibitor may not require whole breast irradiation. A boost is recommended for patients with the following risk factors: age <50 years, lymphovascular invasion, positive nodes, and close margins. A boost may be considered optional for a patient with favorable prognostic factors such as age >60, negative margins, and low grade tumor.

Prospective Randomized Trials supporting the use of breast conservation.

1. Fisher, B., et al. (2002). Twenty-year follow-up of a randomized trial comparing total mastectomy, lumpectomy and lumpectomy plus irradiation for the treatment of invasive breast cancer. *N. Engl. J. Med*. 347, 1233–1241.

2. Veronesi, U., et al. (2002). Twenty-year follow-up of a randomized study comparing breast-conserving surgery with radical mastectomy for early breast cancer. *N. Engl. J. Med*. 347, 1227–1232.

3. Winchester, D. P., et al. (1998). Standards for diagnosis and management of invasive breast carcinoma. *CA Cancer J. Clin*. 48, 83–107.

4. Van Dongen, J. A., Bartelink, H., Fentiman, I. S., et al. (1992). Randomized clinical trial to assess the value of breast-conserving therapy in stage I and II breast cancer: EORTC 10801 trial. *Natl. Cancer Inst. Monogr*. 11, 15–18.

5. Arriagada, R., Le, M. G., Rochard, F., et al. (1996). Conservative treatment versus mastectomy in early breast cancer: patterns of failure with 15 years of follow-up data. Institut Gustave-Roussy Breast Cancer Group. *J. Clin. Oncol*. 14, 1558–1564.

6. Blichert-Toft, M., Rose, C., Andersen, J. A., et al. (1992). Danish randomized trial comparing breast conservation therapy with mastectomy: six years of life-table analysis. Danish Breast Cancer Cooperative Group. *J. Natl. Cancer Inst. Monogr*. 19–25.

Dose selection: most randomized studies have used 5000 cGy in 25 fractions. The NSABP B-39 study randomizing patients to partial breast irradiation verses standard fractionation uses the following doses for the standard arm: whole breast 5000 cGy at 200 cGy per fraction or 5040 cGy at 180 cGy per fractions followed by an electron boost 1000–1600 at 200 cGy per fraction. NCCN recommends a range of doses 45–50 Gy in 1.8–2.0 Gy fractions and boost 10–16 Gy in 2 Gy fractions.

Data for lumpectomy plus tamoxifen without radiation therapy:

1. Hughes, K. S., Schnaper, L. A., Berry, D., et al. (2004). Lumpectomy plus tamoxifen with or without irradiation in women 70 years of age or older with early breast cancer. *N. Engl. J. Med*. 351, 971–977.

2. Fyles, A. W., McCready, D. R., Manchul, L. A., et al. (2004). Tamoxifen with or without breast irradiation in women 50 years of age or older with early breast cancer. *N. Engl. J. Med*. 351, 963–970.

Rationale for using boost and dose:

1. Bartelink, H., Horiot, J. C., et al. (2007). Impact of a higher radiation dose on local control and survival in breast-conserving therapy of early breast cancer: 10-year results of the randomized boost versus no boost EORTC 22881-10882 trial. *J. Clin. Oncol*. 25, 3259–3265.

2. Poortmans, P. M., Collette, L., et al. (2009). Impact of the boost dose of 10 Gy versus 26 Gy in patients with early-stage breast cancer after a microscopically incomplete lumpectomy: 10-year results of the randomized EORTC boost trial. *Radiother. Oncol*. 90, 80–85.

Randomized Trials showing improvement in skin toxicity with the use of IMRT.

1. Pignol, J. P., Olivotto, I., et al. (2008). A multicenter randomized trial of breast intensity-modulated radiation therapy to reduce acute radiation dermatitis. *J. Clin. Oncol*. 26, 2085–2092.

2. Harsolia, A., Kestin, L., et al. (2007). Intensity-modulated radiotherapy results in significant decrease in clinical toxicities compared with conventional wedge-based breast radiotherapy. *Int. J. Radiat. Oncol. Biol. Phys*. 68, 1375–1380.

Dose constraints from RTOG 1005 – a Phase III trial of accelerated whole breast irradiation with hypofractionation plus concurrent boost versus standard whole breast irradiation plus sequential boost for early-stage breast cancer.

QUANTEC references for lung and heart constraints:

Radiation dose-volume effects in the lung. Marks, L. B., Bentzen, S. M., Deasy, J. O., et al. (2010). *Int. J. Radiat. Oncol. Biol. Phys*. 76(Suppl. 3), S70–S76.

Radiation dose-volume effects in the heart. Gagliardi, G., Constine, L. S., Moiseenko, V., et al. (2010). *Int. J. Radiat. Oncol. Biol. Phys*. 76(Suppl. 3):S77–S85.

Breast cancer atlas for radiation therapy planning: consensus definitions http://www.rtog.org/pdf_file2.html?pdf_document=BreastCancerAtlas.pdf

– Uploaded or MOSAIQ
– Hot Scripts created in Pinnacle
– Billing/CPT reviewed
